# Neuroimaging, a new tool for investigating the effects of early diet on cognitive and brain development

**DOI:** 10.3389/fnhum.2013.00445

**Published:** 2013-08-06

**Authors:** Elizabeth B. Isaacs

**Affiliations:** Childhood Nutrition Research Centre, UCL Institute of Child HealthLondon, UK

**Keywords:** early diet, brain development, cognition, neuroimaging, programming

## Abstract

Nutrition is crucial to the initial development of the central nervous system (CNS), and then to its maintenance, because both depend on dietary intake to supply the elements required to develop and fuel the system. Diet in early life is often seen in the context of “programming” where a stimulus occurring during a vulnerable period can have long-lasting or even lifetime effects on some aspect of the organism's structure or function. Nutrition was first shown to be a programming stimulus for growth, and then for cognitive behavior, in animal studies that were able to employ methods that allowed the demonstration of neural effects of early nutrition. Such research raised the question of whether nutrition could also programme cognition/brain structure in humans. Initial studies of cognitive effects were observational, usually conducted in developing countries where the presence of confounding factors made it difficult to interpret the role of nutrition in the cognitive deficits that were seen. Attributing causality to nutrition required randomized controlled trials (RCTs) and these, often in developed countries, started to appear around 30 years ago. Most demonstrated convincingly that early nutrition could affect subsequent cognition. Until the advent of neuroimaging techniques that allowed *in vivo* examination of the brain, however, we could determine very little about the neural effects of early diet in humans. The combination of well-designed trials with neuroimaging tools means that we are now able to pose and answer questions that would have seemed impossible only recently. This review discusses various neuroimaging methods that are suitable for use in nutrition studies, while pointing out some of the limitations that they may have. The existing literature is small, but examples of studies that have used these methods are presented. Finally, some considerations that have arisen from previous studies, as well as suggestions for future research, are discussed.

## Introduction

The fact that the study of nutrition is basic to brain science often goes unrecognized by neuropsychologists and cognitive neuroscientists who tend to regard it as a variable of little or no interest. Most fundamentally, and throughout life, energy to fuel the brain comes from dietary intake, mainly in the form of glucose. Other elements in the everyday diet also influence brain function by providing resources for the maintenance of central nervous system (CNS) activity; Greenwood and Craig (1987), for example, describe some ways in which food intake can affect neurochemistry: by providing precursors for the synthesis of neurotransmitters; by providing vitamins and minerals that serve as essential co-factors in enzyme activity during synthesis; by providing dietary fats that can affect properties of the nerve cell membranes. Variations in neural processes such as these, resulting from dietary intake, may have demonstrable cognitive consequences.

In addition to these ubiquitous effects across the human lifespan, nutrition has an important role to play in the initial development of the brain. Indeed, according to Walker (2005), nutrition is possibly the single environmental variable that can have the widest range of effects on brain development. Nevertheless, it is important to remember that it is one of many other environmental factors such as socioeconomic status, maternal attachment, and level of parental education that play an important role in neural development. Recently, epigenetic factors (i.e., modifications to the genome that do not involve changes in nucleotide sequence) have been shown to be modifiable by environmental factors, making this a candidate mechanism for the mediation of the effects of early experience on the development of the organism (Zhang and Meaney, 2010; Blaze et al., 2013). There are two features of nutrition that emphasize its important role: Rosales et al. (2009) have pointed out that nutrition is unique in that it can directly modify genetic structure and also mediate how genetic factors are expressed. It is also more amenable to modification than many other factors.

One of the hallmarks of developmental neuropathology is the idea that a stimulus will have its greatest effect on the structures and processes that are developing at the time it is applied (Dobbing, 1981). In other words, the CNS is most vulnerable to environmental influence, such as nutrition, when it is in a state of change and plasticity is at its greatest; in the case of the human infant, this period of rapid growth and development, known as the “brain spurt,” extends from the beginning of the third trimester of pregnancy until ~2 years of age (Gilmore et al., 2012). Georgieff (2007) states that the brain is particularly vulnerable to the influences of nutrition between 24 and 42 weeks of gestation. During the growth spurt, the brain follows an invariant sequence of developmental events, beginning with neuronal proliferation and migration, followed by neuronal differentiation, the formation of connective patterns, subtractive processes in the form of neuronal death and synaptic elimination and, finally, myelination (de Graaf-Peters and Hadders-Algra, 2006). By the age of ~2 years, the volume of the human brain has reached 80–90% of adult size (Knickmeyer et al., 2008). This rapid period of growth has become a focus of research into how variation in nutritional intake may affect subsequent brain development. This does not mean, of course, that nutrition cannot exert effects at other times but envisages a continuum across the lifespan with variations in vulnerability at different ages. Many studies have investigated the important role played by maternal nutrition via the placenta on brain development (Morgane et al., 1993). Antonow-Schlorke et al. (2011), for example, reported that even moderate restriction of maternal nutrition during pregnancy, to a degree not uncommon in the developed world, resulted in major cerebral development disturbances in baboons.

There has been a recent explosion of research activity exploring the early origins of adult disease hypothesis (Barker et al., 1993; Lucas et al., 1999) that, in its original form, stated that low growth rates *in utero* and during infancy were associated with high death rates from cardiovascular disease. This has generated increased interest in infant nutrition. Early studies by McCance (1962) are relevant in this context. The size of the litter in which new-born rats spent the first 3 weeks of life was manipulated. Not surprisingly, those reared in small litters, with proportionally more of the mother's milk supply per individual, were heavier and longer at the end of this time than those from larger litters. At this point, all animals were allowed unlimited food but the smaller animals showed no catch-up growth and this persisted throughout life, so that they never attained the size of the larger; in fact, the differential tended to increase with age. A period of under-feeding of the same duration but introduced at 9 weeks in animals that had been developing normally also resulted in smaller size but, in these circumstances, the re-introduction of a normal feeding regime resulted in rapid weight gain until they attained the “normal” size expected. Studies of this type illustrated that an event early in life, during a limited developmental window, could “programme” long-lasting differences, i.e., that nutrition could act as a programming stimulus for growth. While much of the evidence for a link between early growth and adult outcomes has been observational, a series of studies based on randomized controlled trials (RCTs), reported that early nutrition was shown to programme health-related outcomes such as blood pressure (Singhal et al., 2008), obesity (Singhal et al., 2010), and atherosclerosis (Singhal, 2006). These results raise the question of whether early nutrition could also programme cognition and its underlying neural bases.

While many nutrients have a role to play in brain development, certain ones seem to be of particular importance during fetal and neonatal life: protein, iron, zinc, selenium, iodine, folate, vitamin A, choline, and long-chain polyunsaturated fatty acids (LCPUFAs) (Georgieff, 2007). Each of these has generated a research literature examining relationships with cognition/brain, but these will not be reviewed here where the main concern is the role of neuroimaging in nutrition studies. It is interesting to note, however, that Dobbing (1981) has suggested that all nutrient deficiencies exert their influence through a common pathway of growth restriction of the brain in some form.

## The history of nutrition studies

The study of how nutrition early in life might affect cognition is a long-standing area of research but the form that such investigations take has changed dramatically over time. Initially, the emphasis was on determining minimal nutrient requirements and preventing deficiencies, usually in developing countries and often in areas of severe malnutrition (Lucas et al., 2001). These observational studies reported associations between malnutrition and cognitive behavior but their interpretation was confounded by factors such as poverty, poor parental education, and low levels of environmental stimulation that in themselves could affect cognitive development, making interpretation of the role of nutrition difficult.

There was a clear need for RCTs to establish the causative role of nutrition. Although direct extrapolation to humans was not appropriate, the animal literature was suggestive. Smart (1986), for example, reviewed 165 studies, mainly in rodents, examining the effects of early under-nutrition on subsequent cognitive behavior such as maze learning. RCTs of nutrition in early human life began to appear around 30 years ago. The most frequently used supplement in these trials has probably been LCPUFAs and systematic reviews of studies in both term (Simmer et al., 2011) and preterm infants (Schulzke et al., 2011) have recently appeared. Breastfeeding has also generated a lot of interest and while it is not possible to randomly allocate infants to breastfeeding, Kramer carried out a RCT (PROBIT) that assigned mothers to groups that received either extra encouragement to extend the duration of breast feeding or the standard level of advice (Kramer et al., 2008). Lucas carried out pioneering studies with random assignment to either high or low nutrient diets in terms of protein/calorie content in preterm infants (Lucas et al., 1984); Morgane et al. (2002) describe protein as the most critical component of dietary intake for the development of neurological function. This large cohort has been followed up in a series of studies examining the effects of early diet not only on cognition (Lucas et al., 1990, 1998; Isaacs et al., 2009), but also growth (Lucas et al., 1984), cardiovascular function (Singhal, 2006) and bone health (Fewtrell et al., 2009). These RCTs marked a shift in the context of early nutrition research to include studies set in developed, industrialized societies consuming a typical Western diet where lack of calories is not often a problem.

The demonstration of these cognitive effects raised the possibility that nutrition might be affecting the underlying neural substrates either in terms of structure or function. Again, the animal literature was suggestive. Early nutrition was reported to have effects both at the whole brain and cellular level: cortical thickness (Katz et al., 1982), number of neurons (Fish and Winick, 1969), myelination (Dobbing, 1964), and dendritic development (Salas et al., 1974; Cordero et al., 1986) for example. These discoveries were made possible, however, by methods not available for use in living human subjects. Some limited information was provided by human *post-mortem* studies; Benítez-Bribiesca et al. (1999), for example, reported dendritic spine pathology in the brains of infants severely malnourished in early post-natal life. Electroencephalography (EEG) provided some *in vivo* information; breast-fed infants weaned between 4 and 6 months, to either a control or LCPUFA supplemented formula, demonstrated more mature visual evoked potential (VEP) acuity when tested at 1 year if they were in the supplemented group (Hoffman et al., 2003). It was only with the advent of neuroimaging, however, allowing examination of the brain *in vivo*, that these questions could be widely addressed in human subjects. We are entering a new era of research methodology where we can examine the effects of early nutrition at both the behavioral and neural levels.

While neuroimaging held the promise of demonstrating changes in the brain attributable to nutrition, the advent of MRI scans did not allow this initially because of the nature of the effects expected to occur. The introduction of post-natal feeding, even in very preterm infants, does not occur before a gestational age (GA) of 23/24 weeks. By this time, neuronal proliferation and migration are reaching an end and nutritional intervention, therefore, is unlikely to affect these processes. Instead, the subsequent developmental events of synaptogenesis, glial cell proliferation, and myelination are vulnerable. This means that anomalies in brain structure associated with early nutrition are unlikely to be frank lesions (because no tissue has been destroyed) but rather, “quantitative deficits and distortions” (Dobbing, 1985). These more subtle morphometric anomalies occurring at the level of neuronal organization are not detectable by visual inspection of the scans, the usual clinical method of interpretation. Dobbing (1981) pointed out that these diffuse deficits, of serious consequence to society as a whole, would remain difficult (and maybe impossible) to demonstrate in humans. This skepticism was only assuaged when powerful post scan-acquisition processing techniques became available, providing a robust method for investigating subtle differences in the living brain.

## The use of neuroimaging to study the effects of early nutrition

When used in well-designed studies, neuroimaging may be a useful tool in helping to elucidate the effects of dietary intake. For example, breastfeeding in infancy has been shown to be associated with better cognitive outcome later in life, (e.g., Anderson et al., 1999; Drane and Logemann, 2000), although the evidence is not unanimous (Der et al., 2006). It would be informative if we could determine what changes in underlying brain structure were associated with these cognitive differences, with possible practical consequences in the production of infant formula.

While offering the prospect for studying hypotheses generated by both the animal and human literatures, there are certain constraints when using neuroimaging with infants and children that do not apply in adult studies. Although MRI studies are acceptable for use in a pediatric population, because they are non-invasive and do not involve the use of ionizing radiation, scanning demands a degree of immobility in order to avoid movement artifacts and this may not be possible in young children. While sedation is an option in clinical situations, this is not so when scans are being obtained for research. In practice, this restricts the ages at which children can be studied. In the new born and for the first few months after birth, MRI scanning can be carried out during natural sleep following feeding (“feed and wrap”) but then becomes more difficult until the age of four or five. Various techniques such as pretraining in a mock-up scanner and/or playing audio books/music or showing cartoons can be used to try to lower the age of viability but, in reality, MRI does not become a truly reliable option again until early school age. It is unfortunate that the use of MRI is at its most difficult at the very time when the brain is developing most rapidly and when we expect nutrition to be exerting maximum effects. Other imaging techniques, discussed below, can provide useful information during this “silent” period and cognitive/behavioral measures may be obtained. Because of the above restriction, published studies tend to look at long-term effects associated with infant diet; one might argue that effects that persist are of greatest importance anyway.

The most usual form of post-natal nutritional study is the experimental intervention trial when groups differing in the amount or type of nutrient given as a supplement at one time point are then compared on outcome measures at one or more subsequent times. The most easily interpreted of such studies are RCTs and some such studies with both cognitive and MRI outcomes have started to appear in the literature (see below). Observational trials where, for example, we obtain scans and look at relationships with current diet, are also possible but are less informative with regard to causation, especially since nutrition is associated with a wide variety of other variables such as socio-economic status. Two large collaborative studies funded by the European Union (EARNEST and NUTRIMENTHE: www.project-earlynutrtion.eu and www.nutrimenthe.eu) have followed up a large number of experimental studies (but with no original intention to conduct long-term follow-up) and several of these have included neuroimaging (White et al., 2013) with results due to appear in the near future.

## Neuroimaging methods

Although most neuroimaging studies of early nutrition reported so far have used structural (or anatomical) MRI, the only method that is ruled out in children for research purposes is positron emission spectroscopy, because of the radiation involved (although it has been used in clinical situations). A description of the basic methods is given below in case these are not familiar to nutritionists. [For more detailed information see Paus (2010) or ILSI, if published]. A good review of more recent developments in both scanning protocols and analytic techniques is given by Vasung et al. (2013).

### Structural MRI

The choice of MRI method depends on the hypothesis being investigated, as well as the age of the subjects. The main distinction is between “structural/anatomical” scans that image the brain anatomy resulting from genetic and environmental factors up to the point of scanning, and “functional” scans (fMRI) that reflect present physiology, by measuring changing oxygenation levels in the cerebral blood supply. If our interest is in long-term effects of infant diet, then we are more likely to collect structural scans, although early diet could also influence the way the brain processes information as reflected in fMRI. Short-term studies, such as those looking at the immediate effects of breakfasts varying in macronutrient content, are more likely to measure concurrent functional effects.

There are also choices to be made within these categories since there are different MRI imaging protocols available; the choice will depend on what method of data analysis is to be used post-acquisition, and that, in turn, is partly determined by the research hypothesis. In the study of morphometry and volumetrics, the most widely used structural protocol results in a 3D data set of T1 weighted images. MR imaging involves aligning the protons in the nuclei by placing them in a magnetic field and then perturbing that field and, hence, the protons; T1 refers to one aspect of the time (relaxation time) it takes for the nuclei to return to their initial aligned state. These scans are particularly suitable for anatomical analyses because they provide sharp contrast between gray and white matter. Once the scans are acquired, a wide variety of analysis techniques is available. Automated methods such as Freesurfer (Fischl et al., 2002) provide measurements of the volumes of anatomical structures and of other features such as the thickness of the cortex (Fischl and Dale, 2000) or the degree of cortical gyrification in newborns (Dubois et al., 2008). These quantitative data can then be analysed statistically to determine differences between groups or can be correlated with other measures such as cognitive outcomes. Another widely-used technique is voxel-based morphometry (VBM; Ashburner and Friston, 2002), designed to determine local differences in the distribution of gray and white matter in the brain between groups, such as those with different early dietary interventions, which can be compared to determine if there are areas of the brain where they differ in tissue type distribution; correlations between gray or white matter and other measures (e.g., nutrient measures) can be examined.

A more recently developed structural protocol is diffusion tensor imaging (DTI), used to examine aspects of the microstructure of white matter (primarily), often not apparent on standard MRI, by measuring the diffusivity of water molecules in the brain tissue. The brain is anisotropic and water flow is constrained by anatomical features such as the degree of myelination of the axons. Metrics sensitive to various aspects of diffusion are produced; fractional anisotropy (FA), for example, reflects the directionality of water flow, and FA maps can be analysed in similar ways to T1 scans, using VBM to look for differences between groups of scans. DTI can be used to measure both the degree of myelination and also density in unmyelinated nerve fibers. Because nutrition is likely to affect both dendrites and axons, known collectively as neurites, a recently-developed diffusion MRI technique called Neurite Orientation Dispersion and Density Imaging (NODDI; Zhang et al., 2012) may be well-suited for use in early nutrition studies. Another popular voxel-based method of analysis is tract-based spatial statistics (TBSS; Smith et al., 2006), part of the FMRIB Software Library (FSL: www.fmrib.ox.ac.uk). This method creates white matter “skeletons” consisting of voxels common to all scans within a group and then compares these between groups. DTI data can also be used in tractography studies (Feigl et al., 2013) that visually represent the neural tracts and networks underlying functional connectivity by using 3D modeling techniques.

Magnetic Resonance Spectroscopy (MRS) is also based on structural scans but the emphasis here is on measuring concentrations of metabolites in the brain (a wide variety but commonly including aspartate, choline, creatine, glucose, and taurine). It is a non-invasive method for studying the metabolism of tissue *in vivo* (Gadian, 1995). In this MR application, the radiofrequency pulse in the magnetic field picks up signals from different metabolites depending on the nuclei under study. Signals are obtained from a defined region of interest and chemical spectra produced. Changes in the metabolic concentrations measured by these spectra occur over the lifespan and data can be compared with known population values at different stages of life. It has been widely used clinically (e.g., in the study of epilepsy) but, although it would seem to be of particular relevance to nutrition (for example, in studies of phospholipids in neuronal membranes), studies are sparse. Sizonenko et al. (2006) used MRS to measure creatine using MRS in babies with *in utero* growth restriction (IUGR), and showed marked increases at term compared to control infants.

### Functional MRI

Functional studies make use of the fact that brain activity is linked to changes in cerebral blood flow and the level of oxygenation in the blood. Areas of the brain that are active require oxygen and, as a consequence, blood flow increases to these areas; the blood oxygenation level falls as neural activity continues. fMRI uses the different magnetic properties of oxygenated and deoxygenated blood to image areas of brain activity. A study by Akitsuki et al. (2011), although not conducted in children, shows how this method could be used to study the relationship between elements of nutrition and cognitive function in younger populations. Six young adult males took part in a repeated measure, counterbalanced crossover study that used fMRI to study how the nutritional quality of breakfast affected cognitive function and the brain. A nutritionally balanced breakfast produced higher brain activation than either water or a sugar/water mixture in the medial aspect of the prefrontal cortex during the performance of a working memory task. This illustrates how short-term responses to nutrition might be measured but it is also possible that early changes in brain structure due to nutrition may lead to differences in how cognitive tasks are carried out in the long-term.

Originally, fMRI studies involved measuring the brain's response to some external stimulus designed to activate different regions, as in the Akitsuki et al study (2011). More recently, fMRI imaging has been used to acquire images in the absence of external stimulation (resting state) in order to detect brain regions that are highly correlated with each other temporally, described as the brain's default network. This resting fMRI is potentially a useful technique in young children (Fransson et al., 2011; Gao et al., 2011) because they are not required to pay attention nor cooperate in carrying out cognitive tasks. Resting state functional connectivity studies (Knickmeyer et al., 2008; Gao et al., 2009), along with DTI tractography, used in conjunction with new methods of data analysis such as graph theory (Bullmore and Sporns, 2009), are all consistent with current conceptions of brain function as a network where connections between neural areas are of importance (e.g., Jung and Haier, 2007), rather than the older emphasis on modularity of structure. No studies have yet been done in children but early diet might well be seen to impact the nature of the resting state default network and its development.

### Other methods

One of the original techniques for studying brain function is EEG, used to measure the electrical activity of the brain from electrodes placed on the surface of the head. While temporal resolution is good (<1 ms), providing a direct measurement of brain activity, spatial resolution is less so. Magnetoencephalography (MEG) is a more recent development that measures brain electromagnetic activity. Both are non-invasive and easily repeatable as well as being less sensitive to movement than MRI, making them suitable for use with even quite young children. They can be used to record brain activity while carrying out tasks, or measure responses to environmental stimulation (event-related potentials—ERPs), sometimes using paradigms that do not require the child to actively attend (e.g., oddball tasks). They are suitable across the life span for measuring how early intervention might have affected brain processing and also for studying the immediate effects of nutrient intakes (such as caffeine in adults).

Near Infrared Spectroscopy (NIRS), sometimes called optical imaging, is another method suitable for use in children. Like fMRI, it makes use of hemodynamics, but the energy source in this technique is near-infrared laser or LED light shone through the skull. The light is diffused through the upper layer of the cortex and is then absorbed by optodes positioned on the skull. Oxygenated and deoxygenated hemoglobin have different light absorption properties and NIRS makes use of this information to produce measures that correspond strongly to the fMRI BOLD signal (Blood Oxygenation Level-Dependent; see below). Like EEG, temporal resolution is superior to spatial. It has many of the advantages of EEG for use with children although, to date, it has been used mainly in a clinical context. Its advantages, along with recent improvements in the metrics it produces, indicate that it could be a useful tool in the study of early nutrition's effects on the brain and cognition but studies have yet to be reported.

## Early nutrition studies that have used neuroimaging techniques

If we eliminate studies in clinical populations, such as anorexics, relatively few using neuroimaging to examine the effects of early diet have so far been reported in the literature, but that can be expected to change in the future. Although the ultimate aim would be to define dietary intake for optimal brain/cognitive development, the research is still at the “proof of principle” stage.

The most widely used technique has been structural MRI. MRI showed reduced intracranial and cortical gray volumes in infants with IUGR (Borradori Tolsa et al., 2004) compared to preterm infants matched for GA but with appropriate intrauterine growth; there was also a correlation between cortical gray matter volume and a behavioral developmental measure of attention. Scans were obtained soon after preterm birth and again at term equivalent age and it is interesting to note that the IUGR infants showed no catch-up growth in total intracranial and cortical gray matter volumes in this time period (reminiscent of McCance's rats) despite the rapid brain development that is occurring. Lodygensky et al. (2008) used MRI to obtain volume measurements of the hippocampus and demonstrated a reduction in IUGR infants at term age compared to matched non-IUGR infants. In this case, the hippocampal measurements were related to all six domains of a preterm behavioral assessment. Sizonenko et al. (2006), in a study using MRS, reported that babies with IUGR, reflecting deficient prenatal nutrition, showed marked increases in creatine at term compared to control infants. These results clearly demonstrate alterations in brain tissue volumes, both total and regional, and in metabolite concentration in infants who have been growth restricted *in utero*. While nutritional deficiencies no doubt play a role in the genesis of the effects on brain tissue, it is likely that other factors in the stressful intrauterine environment that these infants inhabit are also implicated; it is difficult to disentangle these. At present, preterm infants have a limit of viability (50% survival rate) of around 23–24 weeks GA so post-natal nutrition will always be introduced during the second major phase of brain growth, resulting in differences mainly in white matter. IUGR, however, can occur during the first phase and this may be why we tend to see gray matter differences, rather than white, in such infants.

Several neuroimaging studies have been carried out in adolescents who were members of the original preterm cohort RCT of infant feeding reported by Lucas et al. (1984). All infants were randomly assigned to either a high or low nutrient diet for the duration of their hospital stay. The low nutrient diet was either banked breast milk from donors or the formula that was in standard use at the time, while the high nutrient diet was an enriched formula prepared for the study that contained extra protein and calories (for full details of formulas see Lucas et al., 1998). Mothers were asked if they planned to breastfeed or not. If not, their infants received their randomized diet as their sole feed (100%). If mothers chose to breastfeed, then the assigned formula was used only if supplementary feeding was required; depending on the success of breastfeeding, the percentage of expressed maternal breast milk in the infant's diet could range from 0 to 100% of the total intake (%EBM). Follow-up studies to examine cognitive development took place at 18 months, using the original Bayley Scales of Infant Development (BSID; Bayley, 1969; Lucas et al., 1990) and 7.5–8 years when IQ was measured using the Wechsler Intelligence Scale for Children-Revised UK edition (WISC-R, UK; Wechsler, 1974) (Lucas et al., 1998). In both studies, there were differences in favor of the group that had received the high-nutrient diet. The effects were more marked in boys [as in animals (Smart, 1986)] and, at 7.5–8 years, Verbal IQ (VIQ) showed a greater difference between groups than did Performance IQ (PIQ), a differential often seen in nutrition studies. These cognitive effects have been shown to extend into adolescence (Isaacs et al., 2009) and formed the basis for the generic hypothesis that the impact of early nutrition on IQ would be related to selective growth restriction of specific brain structures. A more focused hypothesis was suggested by the findings of Abernethy et al. (2004) who showed that growth restriction in the caudate nucleus was associated with lowered IQ in preterm subjects; we speculated that nutritional effects might be implicated. The participants chosen to take part in the imaging studies had all been born at or below 30 weeks GA, were neurologically normal (history/examination at 7.5–8 years) and were all attending mainstream schools. The period between their preterm birth and term birth at 40 weeks, a time when the major brain spurt is occurring), was spent *ex utero* in these infants; this exposure to environmental influences, at an early stage of brain development, might be expected to increase their vulnerability to dietary effects.

Full IQ testing was carried out on 76 adolescents (mean age = 15y9m) of whom 38 each had received a high or low-nutrient formula as preterm infants. Conventional multi-slice imaging, as well as MPRAGE volume acquisition, was obtained on a 1.5T Siemens Vision system. Freesurfer was used to obtain volumes for total brain volume (TBV) as well as a series of subcortical gray matter structures including the caudate nucleus and hippocampus. Consistent with prior results, the high nutrient group had significantly higher VIQ than the low-nutrient group (Isaacs et al., 2008). In addition, the mean volumes for all structures were higher in the high-nutrient group but only significantly so for the left and right caudate nuclei. (TBV and age at scan were controlled in these analyses). Further, both caudate nuclei showed strong relationships to VIQ (and not PIQ). Because of gender differences in the cognitive data, preplanned gender analyses were carried out that found that the effect of diet on caudate volume was gender-specific, shown only by males, and that caudate volume was associated with VIQ in males but not in females. While some nutrition studies have reported gender differences, many have not examined this factor, so that the pervasiveness of effects is largely unknown. If we accept that prenatal under-nutrition may be seen as a form of stress, then the animal research in rats demonstrating that the male brain is more vulnerable to the effects of stress becomes relevant (Sizonenko et al., 2006; Zuena et al., 2008; Weinstock, 2011). The finding also reinforces the recommendation by the US National Academy of Sciences that gender should be considered in the design and analysis of studies in all areas and at all levels of biomedical research (Wizemann and Pardue, 2001). While the study by Isaacs et al. (2008) demonstrated relationships between early diet and both brain structure and cognition, it is important not to over-interpret this to mean that the caudate is the only neural structure affected by early nutrition; other aspects of brain structure not examined here may also be vulnerable.

A second study in the same cohort investigated the relationships among breast feeding, neural structure and cognition in a group of 50 adolescents (Isaacs et al., 2010), children of mothers who had chosen to breastfeed and whose EBM% intake, therefore, varied between 0 and 100% (see above). We correlated %EBM with brain volumes and IQ scores; note that in this study there is no comparison of groups but relationships amongst neural structures, a cognitive variable and a dietary variable are explored with correlation. We know that gray and white matter volumes (GMV; WMV) follow different trajectories of development (Toga et al., 2006; Groeschel et al., 2010) and so absolute volumes of these tissues vary by age. Correlation coefficients, however, are independent of absolute values and as long as the ranks of the individuals' volume and EBM% measurements remain constant, the correlation is unchanged. %EBM was correlated significantly with TBV and WMV, left and right, but was not associated with GMV. When TBV was held constant by covarying it (i.e., determining if differences would be found in specific volumes if TBV was the same for everybody, i.e., relative rather than absolute volumes), we found that %EBM was correlated significantly and positively with WMV and significantly but negatively with GMV. We found the same pattern in boys and girls considered separately as in the group as a whole, although correlations between %EBM and WMV were larger in boys than in girls. In this group of preterm infants it appears that increasing the percentage of breast milk in the early diet increases the amount of white matter relative to gray. How is this related to cognitive function? Overall, there was a significant correlation between %EBM and VIQ only. Here, however, boys and girls showed divergent patterns: all three IQ measures [VIQ, PIQ, and FS (Full-Scale) IQ] were significantly related to %EBM in boys but not in girls. There were no differences in IQ scores between boys and girls but it seems that the percentage of breast milk in the infant diet may affect IQ in boys differently than in girls. Finally, we considered the relationship between the neural volumes and IQ scores. The group as a whole showed no relationship between the IQ measures and TBV but both VIQ and FSIQ were related significantly to WMV. In boys, VIQ was related to TBV and, along with FSIQ, to WBV. There were no significant relationships in girls and no correlations with GMV in any group. This finding is in contrast to results reported by Kafouri et al. (2013) who found that duration of exclusive breastfeeding correlated with gray matter rather than white. Their measure of gray matter, however, was cortical thickness in contrast to cortical volume as in the study here and this, along with a different metric for measuring breast milk consumption, may help to explain the difference. The most likely explanation seems to be related to the timing of the nutrition event, since the infants were born at term rather than preterm when different developmental processes are occurring in the brain.

An interesting study by Tan et al. (2008) used a different approach. They point out that children born preterm are not only at risk of later developmental difficulties in attention, literacy, numeracy, and motor coordination, but are also smaller and lighter with lower head circumference compared to their peers. Head circumference is relevant because relationships between it and both TBV and IQ have been reported (Deary, 2012). The aim of the study was to examine the relationships among developmental outcomes, protein/energy intakes during the first 4 weeks of life, and post-natal growth up to term age, using quantitative MRI at 40 weeks GA to examine brain structure. The study took the form of a RCT of hyper-alimentation (diet containing macronutrients above the recommended values) in which 142 infants born before 29 weeks' gestation were randomly assigned to receive either standard or enhanced parenteral/enteral nutrition between weeks 1 and 4. At term age of 40 weeks they were scanned using the “feed and wrap” method (*n* = 65) on a 0.5T scanner and quantitative data were then extracted from the scans. At 3 and 9 months post-term, the infants' neurodevelopment was measured using the BSID-II; 81 were assessed at 3 months and 71 at 9 months. There were no differences between diet groups in the incidence of gross abnormalities seen on MRI nor in TBV and cortical brain volume or in T2 relaxation times, a MRI measure that is known to decrease with maturation. There were, however, significant correlations between TBV and both energy intake (positive) and energy deficit, defined as the difference between actual and recommended daily intakes (negative), across the pooled groups. CBV and T2 times correlated with a measure of head circumference and both volumes correlated with weight and height at 9 months but with neither birth weight nor head circumference. Analyses of the BSID-II data showed that the mental index (MDI) was correlated with length, TBV, and T2 relaxation time after adjustment for confounding factors. The motor index (PDI) was significantly related to weight. Therefore, while neither the growth nor the MRI measures differed after the dietary intervention, important relationships among growth, neurodevelopment, and brain measures were shown across the whole group. The authors conclude by saying that reducing the energy deficit by improving early nutrition in preterms may improve the growth and maturation of the brain. They also suggest that quantitative MRI measures have the potential to predict both mental and motor outcomes.

The above studies looked at outcome at some future point after early intervention. A study by Taki et al. (2010), however, considered brain structure in terms of concurrent diet in a group of healthy children; although not “infant” diet, this study is included in view of the paucity of studies as an illustration of how neuroimaging can be incorporated into studies of nutrition. Given findings of the positive effects of eating breakfast on cognitive function in children (Benton and Jarvis, 2007), the authors looked at the relationships among breakfast staple type (in terms of glycaemic index—GI), GMV and WMV in the brain and IQ in a group of 290 healthy 5–18 year olds. Participants were divided into three groups depending on their habitual breakfast diet: (1) the “rice” group ate boiled white rice, (2) the “bread” group ate white bread, (3) the “both” group ate one or the other on different days. T1 weighted images were acquired on a 3T scanner, and then analysed using VBM. They confirmed their hypothesis that the “rice” group (low GI) would have greater gray matter than the higher GI “bread” group by demonstrating a significantly higher gray matter ratio (GMV divided by intracranial volume) in the former. This group also had significantly larger gray matter volumes in several regions, such as the left superior temporal gyrus (corrected for intracranial volume and other possible confounding factors), The “bread” group had larger white and gray matter volumes in several regions, including the right fronto-parietal region. Cognitively, the “rice” group had a significantly higher Perceptual Organization score (an Index score from the IQ test). The authors conclude that breakfast staple type affects GMV and WMV and cognitive function (possibly because of the GI differences), and that an optimally nutritious diet is important for brain maturation during childhood and adolescence. Correlational studies, of course, report associations between variables and it must be borne in mind that these do not imply causation.

The oldest existing method for studying brain activity directly, EEG, has also been used in several studies of early nutrition. A study by Li et al. (2010) used ERPs and behavioral measures to study how early diet might influence CNS activity. Data were obtained from 130 healthy full-term infants who were divided into three dietary groups: breastfed (*n* = 40); milk-based formula (*n* = 51); soy-based formula (*n* = 39). Measures were obtained at both 3 and 6 months of age; infants had been on the same diet since they were at least 2 months old and until at least 6 months of age. Behavioral development was assessed at both time points using the BSID and feeding information was collected from 3-day feeding records obtained monthly. ERP responses to language sounds, described as a brain measure of information processing, were collected and measures of ERP amplitude and latency were derived. In terms of behavioral development, all three groups of healthy term-born infants were, perhaps not surprisingly, within the normal range. Differences between diet groups were described by the authors as marginal and transient, but suggested some behavioral advantage for the breastfed group early in development. Females generally improved their scores across the study period more than did males. Although significant group differences were found in the ERP measures, the effect sizes were small. Latency effects in the breastfed group were associated with better developmental scores at 6 months, suggesting that neural maturation might have been more advanced at this point. No differences between the two formula types were found on development or brain activity in this study.

Finally, another EEG study investigated the effects of eating breakfast on the efficiency of the neural networks engaged during mental arithmetic tasks in children aged 8–11 years (Pivik et al., 2012). The authors point out that there is a general consensus that a network of both frontal and parietal brain regions is activated when calculation tasks are being carried out. While many factors influence the ability of an individual to carry out these tasks, they express surprise that nutritional status at the time of assessment is not generally considered as one of them. Mental calculation, for example, has been shown to be sensitive to variations in nutrient intake, such as glucose, as well as the interaction of glucose with macronutrients such as proteins and fats (Dye et al., 2000). The study group consisted of 116 healthy children who had all fasted overnight while staying at the research facility prior to completing the experimental protocol the following day. On awakening, all children completed a battery of cognitive tasks, including mental calculation, and were then randomly assigned to either a breakfast or fasting group; the former consumed a standardized breakfast while the latter continued to fast. The children then repeated the cognitive battery; EEG recordings from frontal and parietal electrode placements were made during both testing sessions. Summarizing, the results showed that the efficiency of the neural networks engaged during mental calculation tasks was enhanced in the group who had eaten breakfast compared to the fasting group; those who continued fasting had to expend greater mental effort in undertaking the tasks. Furthermore, this neural activity was associated with a higher number of correct responses on the mental calculation task.

We are clearly in the very early stages of studying the effects of nutrition on brain variables using neuroimaging but the above examples give some indication of what might be done. Perhaps they are best thought of as proof-of-principle papers at the beginning of a research route that aims eventually to document the characteristics of a diet optimal to the brain/cognitive development of individuals.

## Considerations

In view of the findings from the animal literature, as well as what we know about the effects of environmental influences during human brain development, the results so far obtained from neuroimaging studies in nutrition are biologically plausible. Conceptually, it seems fruitful to consider nutrition as an early programming stimulus for the brain and, hence, cognition, much as McCance (1962) did for growth.

Demonstrating causal effects of early nutrition, rather than just associations, depends on obtaining data from randomized, controlled trials. These are easier to conduct in older children and adults where permission can be obtained to randomly assign the participants to different conditions, e.g., nutritional supplements or meals that vary in macronutrient composition. Even here, however, RCT data may not be straightforward to obtain. There are some interesting data concerning cognitive behavior and high-fat diets in rodents (Greenwood and Winocur, 1990; Winocur and Greenwood, 1999), for example, but it would not be possible to randomly assign human subjects to a high-fat diet that could have adverse consequences for health. The difficulties multiply when trying to carry out studies of early diet in infants and young children, where randomly assigning infants to different dietary groups is unethical in most circumstances. Even when a large RCT such as that of Lucas et al. (1984) is conducted, long-term follow-up is subject to high rates of attrition, making interpretation difficult (for discussion of this problem see Fewtrell et al., 2008). The additional constraints imposed when collecting neuroimaging data in young children necessarily restrict the number of studies that can be carried out and will remain a difficulty with this sort of research, although future imaging methods and protocols may make this more viable. Careful study design may also get around these difficulties. The PROBIT study by Kramer et al. (2008) that looked for effects of breastfeeding on cognition by comparing groups randomized to breast feeding encouragement vs. standard care (resulting in greater duration in the former) suggests ways that information (including neuroimaging) can be obtained.

The timing of the stimulus in programming studies is crucially important. The infants in the Lucas cohort were fed their assigned diet only for the duration of their stay in hospital, (a mean of 4 weeks). After discharge, the infants were fed according to the parents' choice. This may seem a short period to have brought about marked effects in brain structure but there are many examples in the animal world where much shorter periods of stimulation, provided that they occur during vulnerable stages of development, cause permanent change. Perhaps the best known example is imprinting, first described by Spalding and elaborated by Lorenz. Birds show life-long following behavior to the first moving visual stimulus they are exposed to during a brief critical period shortly after hatching, adaptive because it is usually the mother. A single dose of phenobarbital administered to a neonatal rat has lifetime effects on the activity of p450 cytochrome mono-oxygenase activity (Bagley and Hayes, 1983) if administered during a short critical period, but the same dose only induces a period of drowsiness at any other time. Exposure to valproic acid in rats has been shown to cause maximum later autistic-like alterations in social behavior if administered on embryonic day 12 (Kim et al., 2011). The term “critical periods” was used to describe these times with strict temporal boundaries during which specific effects on various aspects of animal behavior were observed. In fact, further research indicated that these boundaries were not as invariant as first thought (Michel and Tyler, 2005) In the case of humans, with a protracted period of brain development and complex behaviors, we are unlikely to see such rigidly defined periods and so the use of the term “sensitive period” seems more appropriate (Johnson, 2005). For example, early squint can affect life-long vision by resulting in conditions like amblyopia (Adams and Sloper, 2003) but the period during which the visual areas of the brain are sensitive extends from birth to around 2 years of age but with no strict upper limit. We do not yet know the parameters of the sensitive, period for dietary effects on brain/cognition in humans; this remains to be empirically determined. Defining this period is of extreme importance, however, especially for infant feeding policy, and needs to be prioritized in nutrition research but the difficulties in obtaining ethical approval in those who are normal and healthy have already been pointed out. Developments in neuroimaging technology are occurring all the time and new methods may become available in the future that will solve some of these problems.

The issue of timing is closely related to the course of brain development. As mentioned earlier, the sequence of steps in brain development is invariant but the timing may differ between regions so that one process may be at its peak in one area of the brain while another is at its peak in a different region (Huttenlocher and Dabholkar, 1997). Two outcomes follow from these principles: (1) the *same* stimulus applied at different times (e.g., nutrition at varying gestational ages) might lead to different brain effects because the neural areas at peak development are not the same, (2) *different* stimuli applied at the same time might have the same effect on the brain. Another important timing principle is that many developmental processes in the brain have the opportunity of occurring only at certain chronologically defined times (Dobbing, 1985), and if one process fails to develop optimally, later processes may be affected. If the opportunity for optimal development is missed, then it does not come again and any effects can be permanent, altering the trajectories of both brain and cognitive development. A study by Banich et al. (1990) looked at the cognitive development of children who had suffered damage to one hemisphere of the brain before birth resulting in congenital hemiplegia. Their IQ scores kept pace with healthy controls up to the age of 6 years but from then on, although their cognitive scores continued to increase, they did so at a slower rate than in controls, so that their developmental trajectories diverged. Interestingly, the same did not apply in children in whom the onset of hemiplegia occurred in post-natal life. It might be that the effects of non-optimal nutrition, during a limited period, have similar effects on subsequent development; neuroimaging studies, particularly longitudinal, could be used to delineate the brain regions involved.

The emergence of two types of specific effects of early diet on subsequent brain structure/cognition should be noted as areas for future research. The first is the differences found between girls and boys, where it appears that the brain's vulnerability to nutritional influences, at least in some cases, differs by gander. There are many references in the non-imaging nutrition literature to gender effects, indicating that they should be examined more thoroughly. It is recommended that all future studies be powered to conduct gender group analyses, so that important effects are not cancelled out by looking only at total groups. The second specific effect is that on the verbal aspects of cognitive function (reflected by the VIQ) rather than the non-verbal (PIQ), again widely reported in the nutrition literature (Lucas et al., 1998; Horwood et al., 2001). This is often assumed to be due to cultural factors since these are known to be correlated with intelligence test scores, particularly VIQ. A study by Edmonds et al. (2010), however, indicates that this might be too easy an explanation. They found that the differences in birth weight (reflecting prenatal nutrition) between members of monozygotic twin pairs (twins derived from a single ovum and known as “identical”) were related to difference in their VIQ scores but not PIQ, with a research design that controls, of course, for potentially confounding factors such as socio-economic status, parental IQ, level of parental education, and genetic makeup.

Timing differences may contribute to explaining these specific effects of nutrition. If the timetable of early brain development were different in boys and girls, then the application of a stimulus (nutrition) might have an effect in the group in which a structure was still developing but not in the group where this period had passed. Various aspects of neural development have been shown to differ between males and females both in infancy (Vasileiadis et al., 2009; van Kooij et al., 2011) and later (deBellis et al., 2001; Sowell et al., 2002). Determining this depends on having detailed information about prenatal and early post-natal brain development in boys and girls separately but such data are surprisingly difficult to find. What is needed are detailed descriptions of brain development such as those of Chi et al. (1977), at the level of individual sulci and gyri, but with separate data for boys and girls. Similarly, the specific effects on VIQ might occur if the neural substrates of PIQ were developed very early or very rapidly compared to those subserving VIQ—the latter would remain vulnerable for a longer time and at a different period. Interaction between these two processes could produce differences between genders in specific aspects of cognition.

We pointed out that gender-specific effects could be masked if only the total group is considered, leading to a potentially erroneous conclusion that there was no demonstrable effect of nutrition. A similar situation might arise if only a sub-group of the population is deficient in the nutrient under study and responds to supplementation; a large effect of nutrition in this group could be masked by no effect in the non-responders. These issues are discussed by Benton and Buts (1990) with regard to studies of the effects of vitamin/mineral supplementation on intelligence. Having measures of the status of the individual with regard to the nutrient under study both before and after supplementation (e.g., plasma LCPUFA levels from a blood sample) enables us to determine whether the supplementation worked, and also look at sub-groups in terms of original status to see if the neural effects differ.

Most nutrition studies have focused on two main time periods in the lifespan, infancy/early childhood and old age. The emphasis has been different in both, optimal conditions for development in early life and protection against decline in the elderly, but both have in common that they are times of change in the brain when it is seen as more vulnerable to intervention. Despite the fact that the changes that become apparent in old age may be the culmination of processes that have been occurring throughout the lifespan, this has often been treated as a discrete epoch. Recent findings indicate that another important period of change in brain development, although not as dramatic as the original brain spurt, occurs in adolescence (Ment et al., 2009; Ramsden et al., 2011). This is also an important time nutritionally as adolescents start to make food choices for themselves, independent of the home environment. Since neuroimaging at this time is subject to fewer restraints than in childhood, it is suggested that studies in this age group might be a particularly fruitful focus for research. Nutrition is probably best seen as having an influence on brain and behavior throughout life (Gomez-Panilla, 2008) but with some periods of enhanced vulnerability when the brain is undergoing rapid change.

With the advent of the non-invasive imaging techniques described here, our knowledge of how the young brain develops in both normal and abnormal conditions has increased. Vasung et al. (2013) describe what they consider to be the three major aspects of this structural development. One is the series of dynamic changes that take place in the thickness of the cortex over development. Shaw et al. (2008), for example, describe both thickening and thinning of the cortex over time and demonstrate different thickness patterns in groups with varying IQ; those with superior IQ at young adulthood had thinner cortices than those in other groups (a useful warning that interpreting brain findings must not be based on naïve assumptions such as bigger always means better). Second is the progressive folding of the cortex that takes place, resulting in complex patterns of gyri and sulci. The third aspect is the establishment of patterns of connectivity between brain regions that underlie functional networks, both intrinsic (resting state fMRI) and those that develop with experience. Individual differences in cognition may be related to individual differences in these dimensions of brain structure. Studies focused on how early nutrition affects these different aspects of development would be of great interest.

Neuroimaging studies can add substantially to our body of knowledge about the early effects of nutrition but they must be used cautiously and interpreted with care. The novelty of neuroimaging has led to a plethora of studies outside the field of nutrition, including some whose conclusions seem ill-founded. It cannot be stressed enough that the most complex neuroimaging techniques available are no substitute for good research design—it is all too easy to be dazzled by the technology. Even the oldest methods are still relatively new and there is continuing discussion about what how some of the measures, in wide use, should be interpreted, e.g., the BOLD signal in fMRI (Logothetis and Wandell, 2004). Some have questioned the validity of post-acquisition analysis such as VBM (Bookstein, 2001). There are statistical considerations with voxel-based methods entailing as they do very large numbers of computations, making adequate correction essential. Although automated procedures have generally replaced manual ones for volumetric measurements, these should not be used uncritically since they are sometimes prone to error. Not all of the neuroimaging measures used have been adequately validated as nutritional markers. And, as pointed out above, interpretation of the brain findings must not be based on naïve assumptions.

Relating cognitive outcomes to the brain findings adds another dimension of possible error. Although there has been a tendency in the past for nutrition studies to measure only IQ, this is short-sighted and may miss important effects. IQ is a complex, composite measure and the same overall values can be arrived at by very different combinations of underlying sub-test scores. In addition, groups with the same IQ score may show marked differences in specific cognitive abilities. Too often, it is concluded that nutrition has no effects because the IQ scores between groups do not differ but specific cognitive effects cannot emerge if they are not measured. A study that looks at the effect of one nutrient can only come to selective conclusions regarding the effects of that particular nutrient on the brain/cognition. In the breastfeeding study described above, for example, it would be incorrect to conclude that the female brain is not vulnerable to the effects of nutrition; we can conclude that the effects of breast milk on the brain are more pronounced in boys and that their breast milk intake is related to IQ in a way not seen in females, but studying another nutrient might lead to gender-specific effects in females.. Negative brain findings are sometimes attributed to the fact that the imaging methods available at present are not sensitive enough to demonstrate effects; this may be true but sometimes the failure to demonstrate effects means that there are … no effects.

The number of studies so far has been small largely because many centers have not had both nutrition and scanning data, but this is changing and a significant number of reports are expected in the near future. The advent of neuroimaging has allowed us to answer questions that would have been difficult to pose even relatively recently. As long as we employ neuroimaging sensibly and responsibly we should expect many more insights into the effects of early diet on the brain and cognition to emerge.

### Conflict of interest statement

The author declares that the research was conducted in the absence of any commercial or financial relationships that could be construed as a potential conflict of interest.
